# Overexpression of Protochlorophyllide Oxidoreductase C Regulates Oxidative Stress in Arabidopsis

**DOI:** 10.1371/journal.pone.0026532

**Published:** 2011-10-21

**Authors:** Gopal K. Pattanayak, Baishnab C. Tripathy

**Affiliations:** School of Life Sciences, Jawaharlal Nehru University, New Delphi, India; George Mason University, United States of America

## Abstract

Light absorbed by colored intermediates of chlorophyll biosynthesis is not utilized in photosynthesis; instead, it is transferred to molecular oxygen, generating singlet oxygen (^1^O_2_). As there is no enzymatic detoxification mechanism available in plants to destroy ^1^O_2_, its generation should be minimized. We manipulated the concentration of a major chlorophyll biosynthetic intermediate i.e., protochlorophyllide in Arabidopsis by overexpressing the light-inducible protochlorophyllide oxidoreductase C (PORC) that effectively phototransforms endogenous protochlorophyllide to chlorophyllide leading to minimal accumulation of the photosensitizer protochlorophyllide in light-grown plants. In *PORC* overexpressing (*PORCx*) plants exposed to high-light, the ^1^O_2_ generation and consequent malonedialdehyde production was minimal and the maximum quantum efficiency of photosystem II remained unaffected demonstrating that their photosynthetic apparatus and cellular organization were intact. Further, *PORCx* plants treated with 5-aminolevulinicacid when exposed to light, photo-converted over-accumulated protochlorophyllide to chlorophyllide, reduced the generation of ^1^O_2_ and malonedialdehyde production and reduced plasma membrane damage. So *PORCx* plants survived and bolted whereas, the 5-aminolevulinicacid-treated wild-type plants perished. Thus, overexpression of *PORC* could be biotechnologically exploited in crop plants for tolerance to ^1^O_2_-induced oxidative stress, paving the use of 5-aminolevulinicacid as a selective commercial light-activated biodegradable herbicide. Reduced protochlorophyllide content in *PORCx* plants released the protochlorophyllide-mediated feed-back inhibition of 5-aminolevulinicacid biosynthesis that resulted in higher 5-aminolevulinicacid production. Increase of 5-aminolevulinicacid synthesis upregulated the gene and protein expression of several downstream chlorophyll biosynthetic enzymes elucidating a regulatory net work of expression of genes involved in 5-aminolevulinicacid and tetrapyrrole biosynthesis.

## Introduction

Chlorophylls (Chls), the most abundant tetrapyrroles in plants, absorb light energy and convert it into chemical energy by the process of photosynthesis. The excess light energy absorbed by Chls, not utilized in photosynthesis, causes an accumulation of excited states of Chls (^1^Chl*) that may subsequently convert to triplet-excited states of Chls (^3^Chl*), which can transfer their energy to molecular oxygen (O_2_). The resulting form of O_2_ i.e., ^1^O_2_ [1], one of the several reactive oxygen species, could cause photooxidation of membrane lipids resulting in photooxidative damage to plants. In excess light, not only Chls, but their colored biosynthetic tetrapyrrolic intermediates i.e., protoporphyrin IX (Proto IX), Mg-protoporphyrin IX (Mg-Proto IX), Mg-protoporphyrin IX monoester {MP(E)}and protochlorophyllide (Pchlide) produce ^1^O_2_ in light-grown plants and cause oxidative damage [2]–[11]. Therefore, to prevent ^1^O_2_-induced oxidative damage in light-grown plants during the daytime it is essential to minimize the steady state concentration of Chl biosynthetic intermediates synthesized from 5-aminolevulinicacid (ALA). ALA is metabolized to Proto IX, MP(E) and Pchlide. Pchlide is one of the major Chl biosynthetic intermediates that accumulate during night [12], [13] and if produced in excess, all can not bind to available limited protochlorophyllide oxidoreductase (POR) [14] proteins and NADPH to form the ternary complex and the prolamellar body formation. The excess unbound Pchlide provokes cell death in light by producing ^1^O_2_ endogenously [5], [8], [15]. There is no enzymatic means available for plants to detoxify ^1^O_2_. Carotenoids prevent the generation of ^1^O_2_ non-enzymatically by quenching the triplet excited states of Chls in the photosynthetic apparatus [16]. However, the ^1^O_2_ produced from Chl biosynthetic tetrapyrrolic intermediates is not effectively quenched by carotenoids because they are not associated with light-harvesting chlorophyll-protein complexes and hence are not connected to the reaction centre [17]. Recently, a few attempts have been made to reduce ^1^O_2_-mediated damage in Arabidopsis or in Chlamydomonas [18]–[20]. These approaches strive to limit the injury to plants after ^1^O_2_ is produced. However, under different stressed environment it is necessary to limit the light-mediated ^1^O_2_ generation from the Chl biosynthetic tetrapyrrolic intermediates in green plants in order to protect them from oxidative damage.

The Pchlide that accumulates overnight is photoconverted to chlorophyllide (Chlide) at day-break by a light-dependent enzyme POR [14]. There are three isoforms of POR (PORA, PORB and PORC) present in Arabidopsis [21]–[25]. The expression of PORA rapidly declines within hours of illumination of etiolated seedlings; PORB expression also reduces significantly after few more hours of light treatment [21], [26], whereas the PORC expression is induced by light [22]–[23], [27]. More interestingly, PORC transcript and protein abundance increases in response to increase in light-intensity and are predominantly present in fully matured light-grown green tissues [27].

The goal of the present investigation is to minimize the generation of ^1^O_2_ by reducing the steady state concentration of the photosensitizer Pchlide in the cells of high-light-grown plants. This is achieved via a genetic approach i.e., overexpression of PORC (*PORCx*) that could efficiently photo-transform Pchlide to Chlide in high-light-grown plants. We show that over-expression of PORC in *Arabidopsis thaliana* reduces the accumulation of Pchlide in high light-grown plants that results in minimal generation of ^1^O_2_ and plants are protected from ^1^O_2_-mediated oxidative damage caused by high light. Further, PORC overexpression could protect the plants from oxidative herbicidal action of ALA. We also show that overexpression of *PORC* results in coordinated upregulation of gene/protein expression of several Chl biosynthetic pathway enzymes resulting in enhanced Chl synthesis in light-grown plants.

## Results

### PORC-overexpressing Arabidopsis leaves exhibited greener phenotype

**Figure 1:**
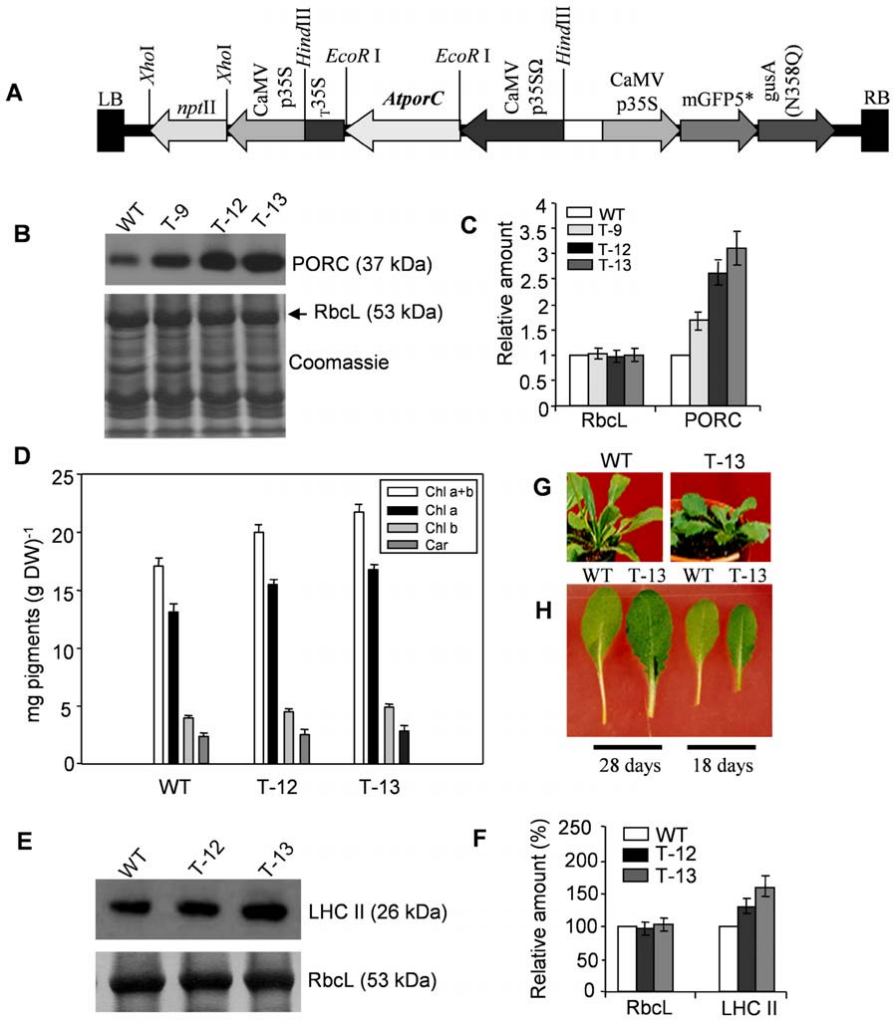
Transformation of Arabidopsis by using *AtPORC* cDNA. (**A**) TDNA region of modified pCAMBIA1304 - 35SΩ *AtporC*. (**B**) Western blot of PORC in WT and *PORCx* plants (T-9, T-12, T-13). Thylakoids were isolated from WT and *PORCx* plants grown for 4-weeks under 14 h L/10 h D photoperiod (100 µmoles photons m^−2^ s^−1^) at 22°C±2°C and thirty µg of thylakoid proteins were loaded in each lane of SDS-PAGE. Western blot was done using PORC (1:1000) monoclonal antiserum (top panel). The bottom panel shows coomassie-stained gel for equal loading. Before coomassie staining, the membrane was probed with RbcL antibody (1:20000) and the respective protein was identified by Western blot. (**C**) Quantification of band intensities of PORC and Ribulose-1, 5-bisphosphate carboxylase oxygenase large subunit (RbcL) presented in ‘B’. (**D**) WT and *PORCx* plants were grown as mentioned above and their chlorophyll and carotenoid contents were measured. (**E**) Western blot of LHC II (1:5000) in WT and T-12, T-13 plants (upper panel). RbcL protein was identified by Western blot analysis as described above and was shown in the bottom panel to check for the equal loading of the thylakoid proteins. (**F**) Quantification of band intensities of LHCII with the RbcL control presented in ‘E’. Signal intensity for each protein was expressed relative to WT. All the above experiments were repeated three times and each data point is the average of three replicates and the error bar represents SD. (**G**) Phenotypic differences of Arabidopsis WT and T-13 plants after 4-weeks of growth at 22°C±2°C under 14 h L/10 h D photoperiod (100 µmoles photons m^−2^ s^−1^). As the T-13 plant has higher PORC amount and higher Chl content, we have only shown its picture. Notice the T-13 plants were greener and slightly smaller than the WT plants. (**H**) Leaves were excised from 18 days old and 28 days old WT and T-13 plants and photographed.

We generated transgenic Arabidopsis plants overexpressing *PORC* cDNA (*PORCx*) under the control of the cauliflower mosaic virus (CaMV) 35S promoter having omega (Ω) translational enhancer (Figure 1A). Protein levels of PORC in four-week-old WT and *PORCx* (T9, T12, T13) plants were analyzed using the PORC monoclonal antibody (Figure 1B). The *PORCx* lines had higher protein expression (1.5–3 folds) of the trans-gene (Figure 1C). They had increased Chl content i.e., up to 28% over that of WT plants and had also a little higher carotenoid content (Figure 1D). The *PORCx* plants/leaves look greener than WT plants (Figure 1G, H). LHC II protein was increased in *PORCx* plants by 33% and 58% respectively (Figure 1E, F).

### PORC expression modulated gene expression and protein abundance of enzymes involved in chlorophyll biosynthesis

**Figure 2:**
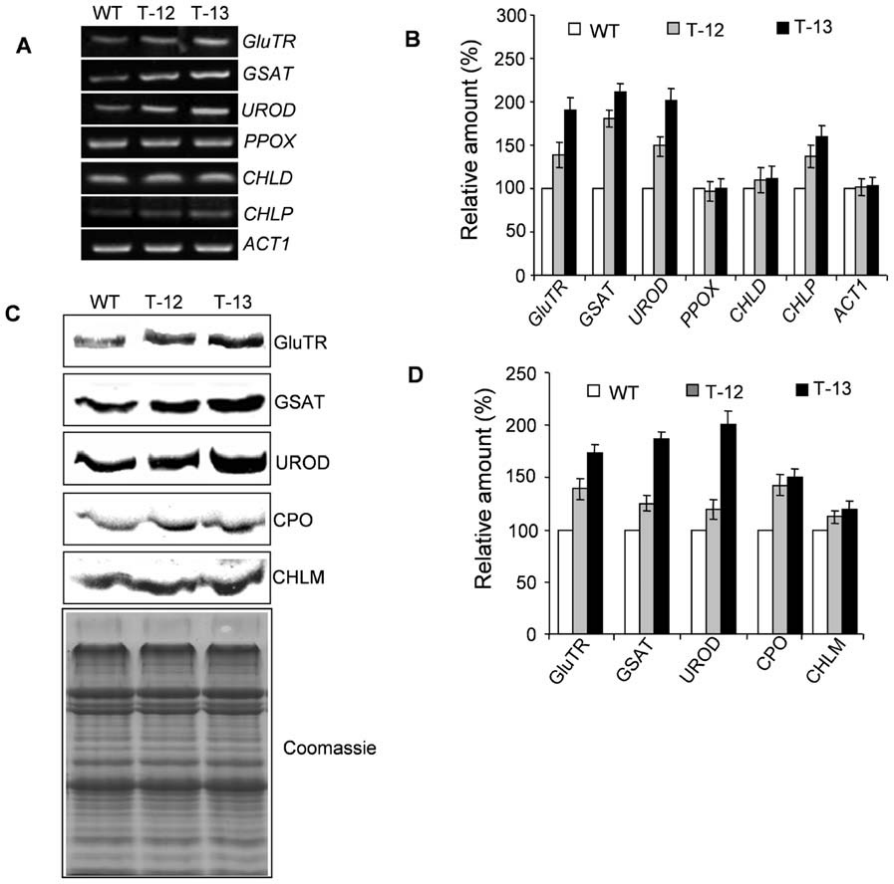
RT-PCR and Western blot analysis of Chl biosynthetic enzymes in *PORCx* plants. (**A**) The gene expression study of the Chl biosynthetic pathway enzymes in T-12 and T-13 plants was done by semi-quantitative RT-PCR analysis using the gene specific primer pairs for each enzyme as described in materials and methods. *ACT1* was used as an internal control. Fifteen µl of the PCR products were loaded and separated on 1% agarose Tris-acetate EDTA gel. Ethidium bromide-stained PCR products were quantified using the Alpha Imager 3400. (**B**) Bar diagram of gene expression (%). Rate of expression is represented as percentage of control (WT). The data presented are representative of three independent experiments. (**C**) Immunoblot analysis of Chl biosynthetic pathway proteins was carried out using plastid proteins isolated from WT and T-12, T-13 plants. (**D**) Bar diagram of protein expression (%) of different enzymes and the rate of expression is represented as percentage of control (WT). Each data point is the average of three replicates and the error bar represents SD.

The increased amount of Chl in *PORCx* led us to study if the over-expression of *PORC* modulated the gene expression and protein abundance of other Chl biosynthetic pathway enzymes. We performed semi-quantitative reverse transcription (RT)-PCR and found the transcript abundance of *GluTR*, *GSAT*, *UROD* and *CHLP* increased respectively by 38%, 80%, 67% and 36% in T-12 and 90%, 110%, 130% and 60% in T-13 *PORCx* lines (Figure 2A, B).

To understand the correlation between the gene expression and protein abundance of Chl biosynthetic enzymes, the Western blot analysis of a few Chl biosynthesis enzymes was performed (Figure 2C, D). As compared to WT, the protein abundance of GluTR, GSAT, UROD and CPO were enhanced, respectively, by 40%, 25%, 20% and 42% in T-12 and 72%, 87%, 111% and 50% in T-13 *PORCx* plants.

### *PORCx* plants have higher POR activity and reduced chlorophyll intermediates in light

**Figure 3:**
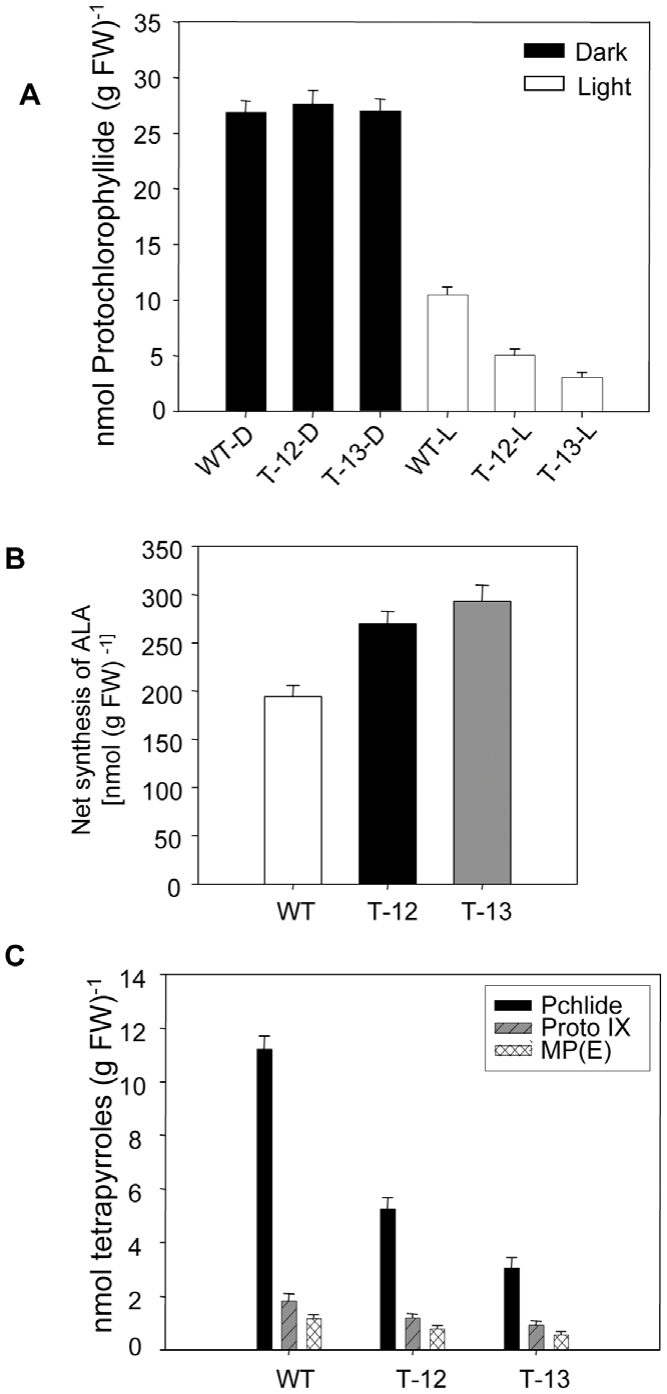
POR activity, ALA content and steady state Chl biosynthesis intermediates of WT and *PORCx* plants. (**A**) Photoperiodically (14 h L/10 h D) grown 4-week-old WT and *PORCx* (T-12, T-13) plants were incubated in dark for 14 h and their protochlorophyllide (Pchlide) contents were determined in dark. After dark incubation, plants were exposed to light (100 µmoles photons m^−2^ s^−1^) for 10 min and their Pchlide contents were monitored and phtotransformation of Pchlide to chlorophyllide was determined. (**B**) Net accumulation of ALA from endogenous substrates of leaves harvested from WT and *PORCx* (T-12, T-13) plants. (**C**) Steady state tetrapyrrole contents of WT and *PORCx* plants. Leaf samples were harvested from photoperiodically (14 h L/10 h D) grown plants during the light phase (7 h after beginning of light cycle), homogenized immediately in light and the chlorophyll biosynthetic tetrapyrroles (Pchlide, Proto IX and MP(E)) contents were estimated. The experiments were repeated for 3 times and each data point is the average of 6 replicates. The error bar represents ± SD.

To measure the PORC activity, four-week-old light-grown WT and *PORCx* plants were kept in dark for 14 h followed by 10 min light exposure (100 µmoles photons m^−2^ s^−1^) and then the photo-transformation of Pchlide was measured. Although a single saturating flash illumination is quite efficient for the transformation of photo-transformable Pchlide pool to Chlide in etiolated tissues, it is not sufficient in light-grown green tissues, for the conversion of non-photo- transformable Pchlide. Actually, exposure of plants to light for several minutes/hours was used before to monitor the disappearance of Pchlide [2], [5], [7], [28]. After 10 min light exposure the Pchlide content was reduced to a greater extent in *PORCx* plants compared to WT plants; the percentage of phototransformation in WT was 61%, whereas in *PORCx* T-12 and T-13 plants it was 82% and 89% respectively (Figure 3A).

Efficient photo-transformation of Pchlide to Chlide in *PORCx* plants decreases Pchlide contents and consequently releases the feed back inhibition of ALA biosynthesis. This resulted in increased ALA synthesis in light-exposed *PORCx* plants (Figure 3B). The extent of overnight Pchlide accumulation by both WT and *PORCx* plants before the end of dark period was quite similar (Figure 3A) although the latter had a greater Chl biosynthesis potential (Figure 2A, C); this is because of feed-back inhibition of ALA synthesis by Pchlide [29], [30]. Due to their enhanced Chl biosynthesis potential, the rate of synthesis of Pchlide and its binding to POR in dark is likely to be higher in *PORCx* plants than that of WT. Once certain amounts of Pchlide accumulate either in WT or in *PORCx*, it would exert the feed-back inhibition on ALA synthesis and consequently, its own synthesis would be down regulated. Because of the efficient assembly of POR-Pchlide complex due to increased abundance of POR protein, the feed-back down regulation of synthesis of ALA and consequently that of Pchlide may occur faster in *PORCx* plants than that of WT. However, prolonged i.e., overnight accumulation of Pchlide would be similar both in WT and *PORCx* plants. Enhanced POR activity as well as increase in gene and protein expression of several Chl biosynthesis enzymes in *PORCx* plants resulted in efficient conversion of Chl biosynthetic intermediates (Proto IX, MPE and Pchlide) to Chl leading to their decreased accumulation in *PORCx* (T-12 and T-13) plants under steady state illumination (Figure 3C).

Since T-13 line had higher Chl content, gene and protein expression, POR activity and lower tetrapyrrole accumulation under steady-state illumination than that in T-12, the T-13 line was further characterized in detail.

### *PORCx* plants are resistant to high light

PORC expression increases with increasing light intensity [23], [27]. To understand the significance of increased expression of PORC in high light, T-13 plants were exposed to high light (350 μmoles photons m^−2^ s^−1^, 16 h light/8 h dark) as described in experimental procedures to know if it could protect plants from light-stress.

### Plant morphology and their photosystem II quantum yield (Fv/Fm)

**Figure 4:**
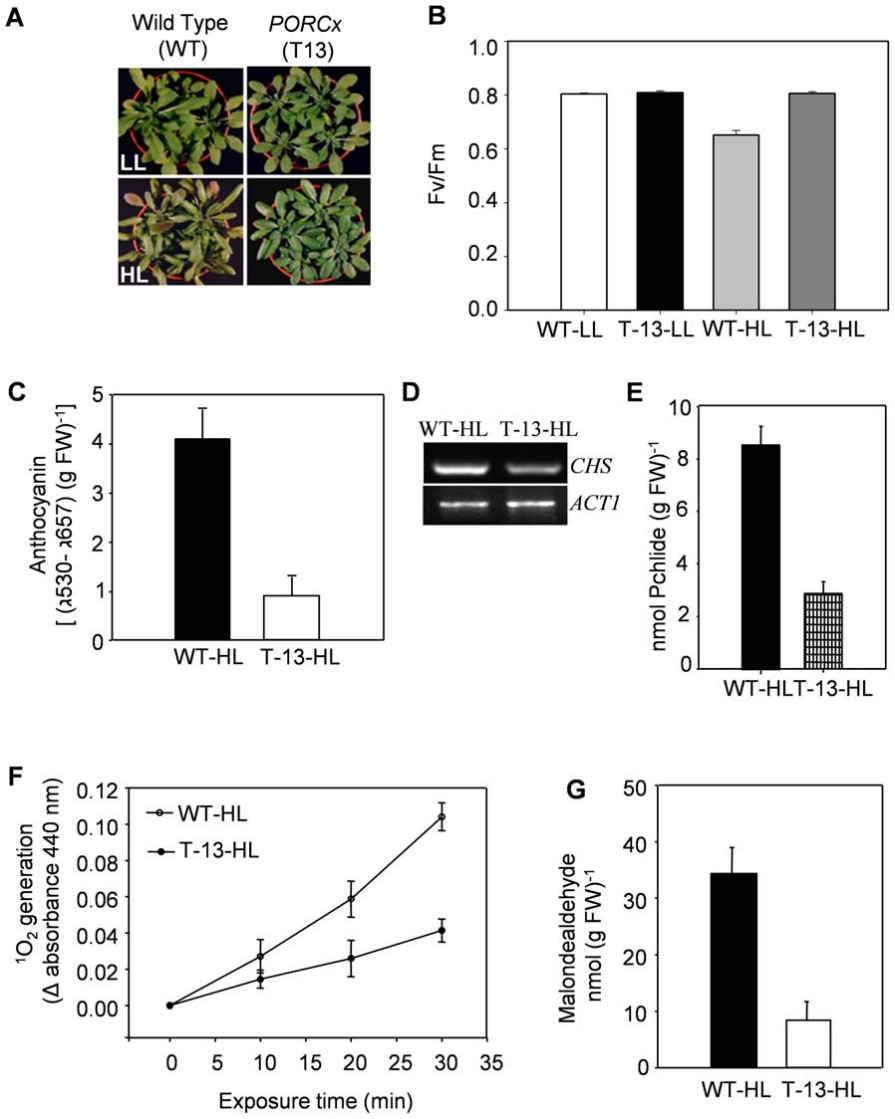
Morphological and physiological responses of WT and *PORCx* plants to light stress. Both WT and *PORCx* (T-13) plants were grown in light (100 µmoles photons m^−2^ s^−1^) for 22–24 days and subsequently transferred to low-light (LL) (50 µmoles photons m^−2^ s^−1^, 16 h light/8 h dark) or high-light (HL) (330 µmoles photons m^−2^ s^−1^, 16 h light/8 h dark) regimes for 6-7 d as described in experimental procedures. (**A**) Photographs of WT and T-13 plants after 6–7 d of transfer to LL and HL. (**B**) Photosynthetic efficiency (Fv/Fm) of leaves of LL- and HL- exposed plants was monitored by PAM 2100 fluorometer. Values are mean ± SD (n = 20). (**C**) Anthocyanin contents of WT and T-13 plants grown under HL. (**D**) The gene expression study of *CHS* in HL-grown WT and T-13 plants was done by RT-PCR as described in experimental procedures. *AtACT1* was used as an internal control. (**E**) Pchlide contents of HL-treated WT and T-13 plants measured 10 min after the end of dark period. (**F**) Singlet oxygen (^1^O_2_) contents in WT and T-13 plants. Thylakoid membranes were isolated in complete darkness from HL- exposed plants and the ^1^O_2_ production was determined in terms of RNO bleaching using histidine as a trap. (**G**) Malondealdehyde (MDA) production in HL- treated WT and T-13 plants. Each data point represented in all the above experiments is the average of 6 replicates. The error bar represents SD.

After 6–7 days of low light (LL; 50 μmoles photons m^−2^ s^−1^) exposure there was no significant difference in the phenotype of WT and T-13 plants (Figure 4A). However, in high light (HL; 350 μmoles photons m^−2^ s^−1^) several leaves of the WT plants looked purple (Figure 4A).

Chl *a* fluorescence is a signature of photosynthetic reactions. The ratio of variable fluorescence (Fv) to maximum fluorescence (Fm) is a measure of quantum yield of photosystem II (PS II) [31]. To understand if light-stress affects photosynthesis, the Fv/Fm ratio was monitored in WT and T-13 plants. The minimal fluorescence F_0_ was not affected by LL or HL. In LL, the Fv/Fm ratio of both WT and T-13 plants was identical (0.8) demonstrating that not only plant morphology but also their photosynthetic efficiency was similar. In HL-stress, the Fv/Fm ratio was substantially reduced to 0.65 in WT plants indicating the quantum yield of PSII was severely affected. Under identical conditions in T-13 plants, the Fv/Fm ratio was normal (0.8) (Figure 4B) demonstrating that PORC overexpression protected plants from HL.

### Anthocyanin accumulation, chalcone synthase expression, ^1^O_2_ production, pchlide accumulation and malondialdehyde content

Under stressful conditions, particularly in high light stress, plants accumulate anthocyanin. As compared to *PORCx* plants, WT plants accumulated higher amounts of anthocyanin (Figure 4C) and as expected the gene expression of chalcone synthase (*CHS*), one of the enzymes involved in anthocyanin biosynthesis, was higher in WT plants as compared to *PORCx* (Figure 4D).

*CHS* expression changes in response to ^1^O_2_ generation [32]. To understand if increased *CHS* expression in HL-treated WT plants is associated with the generation of ^1^O_2_, the latter was monitored by RNO (N, N-dimethyl-p-nitrosoaniline) bleaching reaction [5]. ^1^O_2_ generation was higher in thylakoids of WT plants than that in T-13 plants exposed to light-stress (Figure 4F). Reduced ^1^O_2_ production in HL-stressed T-13 plants was due to reduced steady state accumulation of the photosensitizer Pchlide (Figure 4E). Malondialdehyde (MDA) is an index of lipid peroxidation that is one of the phototoxic consequences of ^1^O_2_-mediated oxidative stress. Reduced ^1^O_2_ generation in light-stressed- T-13 plants resulted in reduced MDA production than that in WT (Figure 4G).

### PORC overexpression protects plants from oxidative herbicidal action of ALA

**Figure 5:**
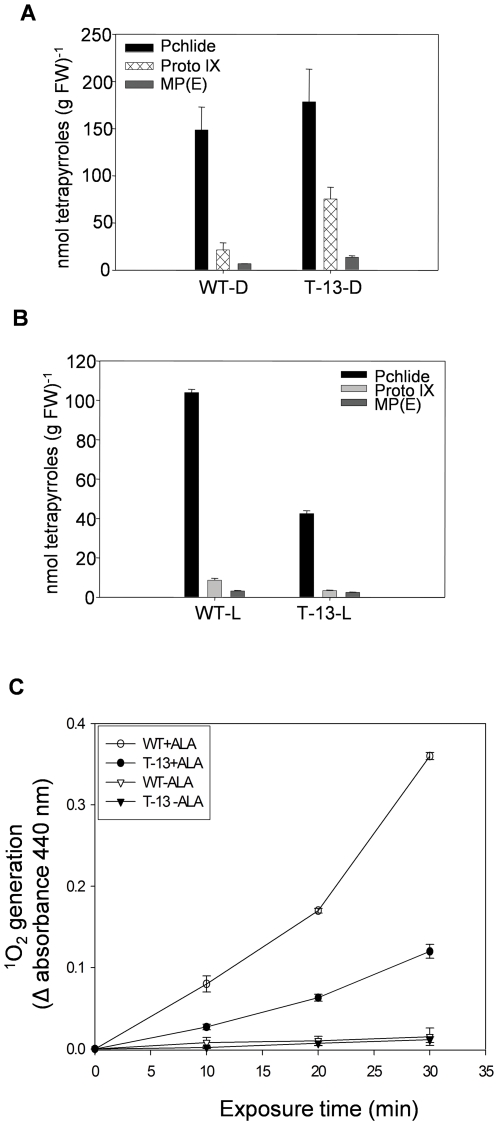
Chlorophyll biosynthetic pathway intermediate contents and ^1^O_2_ production in ALA-treated WT and *PORCx* plants. WT and T-13 plants grown for 28–32 days at 22°C±2°C under 14 h L/10 h D photoperiod (100 µmoles photons m^−2^ s^−1^) were sprayed with ALA (3 mM), dark incubaed for 14 h and exposed to light (100 µmoles photons m^−2^ s^−1^) for 10 min. Leaves were harvested both from dark incubated and light exposed plants, homogenized and their tetrapyrrole contents were monitored by spectrofluorometrically. (**A**) Pchlide, Proto IX and MP(E) contents of ALA-treated (3 mM) and 14 h-dark-incubated WT and T-13 plants. (**B**) After dark incubation both WT and T-13 plants were exposed to light (10 min) and their Pchlide, Proto IX and MP(E) were determined. (**C**) ^1^O_2_ contents in ALA-treated (+ALA) and untreated (-ALA) WT and T-13 plants. The experiments were repeated 5 times and each data point is the average of 5 replicates. The error bar represents ± SD.

ALA is the precursor of Pchlide, which acts like a herbicide in presence of light [2], [4]. Exogenous application of 3 mM ALA to both WT and *PORCx* plants (28-32 days old plants grown under 14 h light/10 h dark photoperiod) at sunset resulted in overnight (14 h) over-accumulation of Chl biosynthetic intermediates i.e., Pchlide, Proto IX and MP(E) (Figure 5A). Pchlide regulates its own accumulation via feedback inhibition of ALA biosynthesis [29], [30]. Treatment of plants with ALA bypasses this regulatory site. Therefore, the feed-back regulation of ALA synthesis by Pchlide is ineffective in the presence of exogenous ALA. In contrast to data reported in Figure 3A, where overnight Pchlide accumulation in non-treated WT and *PORCx* plants is almost equal (because of tight feedback regulation of ALA synthesis by Pchilde), the ALA-treated transgenic plants accumulated higher amounts of Pchlide than that of WT as ALA treatment bypasses the feedback regulatory site and *PORCx* plants have higher Chl biosynthesis potential, as revealed by higher gene and protein expression of their Chl biosynthetic pathway enzymes. Angiosperms are incapable of converting Pchlide to Chlide in dark. Therefore, only after 10 min of light (100 µmoles photons m^−2^ s^−1^) exposure, the Pchlide content of both WT and T-13 plants declined due to light-dependent transformation of Pchlide to Chlide (Figure 5B). However, Pchlide content of ALA-treated and light exposed T-13 plants were significantly lower than that of WT (Figure 5B). This was due to higher efficiency of phototransformation of Pchlide in *PORCx* plants (76%) as compared to that for WT (30%).

### Reduced tetrapyrrole accumulation in *PORCx* plants resulted in decreased ^1^O_2_ production

The generation of ^1^O_2_ from the thylakoid membranes (both dark and light exposed samples) was almost linear till 30 min of light exposure in thylakoid membranes isolated from ALA-treated (3 mM) WT as well as T-13 plants. However, due to reduced accumulation of the photosensitizers Pchlide and other Chl biosynthetic intermediates in light-exposed *PORCx* plants the generation of ^1^O_2_ was nearly half of that of WT (Figure 5C). In untreated (-ALA) plants, due to highly reduced presence of Chl biosynthetic intermediates, ^1^O_2_ generation was much smaller both in WT and T-13 plants.

### ALA-treated plants did not over-produce excess super oxide (O_2_^-^)

It is essential to ascertain if in addition to ^1^O_2_, O_2_^-^ was also generated in light-exposed ALA-treated plants. Tetrapyrroles could produce O_2_^-^ via type I photosensitization reaction where the triplet sensitizer could directly react with the substrate to generate O_2_^-^ [1]. Therefore, O_2_^-^ accumulation was studied in dark and light exposed thylakoid membranes isolated from control and ALA-treated WT plants by cytochrome C reduction assay as described in methods. As shown in Table 1, the amount of O_2_^-^ generated by thylakoids isolated from control and ALA-treated plants was almost similar. The O_2_^-^ produced by the control and treated sample in light was abolished by DCMU (3-(3, 4-dichlorophenyl)-1, 1-dimethylurea), an inhibitor acting at the reducing side of PSII of the photosynthetic electron transport chain. This suggests that the production of O_2_^-^ in control and ALA-treated samples had its origin mostly from the photosynthetic electron transport chain. Mehler reaction is mostly observed in isolated thylakoid membranes at the acceptor side of photosystem I (PS I) [33] that generates O_2_^-^ using H_2_O as the source of electron. Therefore, addition of DCMU that blocks H_2_O oxidation by PS II blocked the generation of O_2_^-^ via Mehler reaction in PSI. These experiments demonstrate that type I photosensitization reaction of plant tetrapyrroles is not involved in O_2_^-^ generation in light-exposed ALA-treated plants and most O_2_^-^ generated in control or ALA-treated plants had their origin from the electron transport chain.

**Table 1:** Production of O_2_^-^ in thylakoid membranes isolated from control and ALA-treated WT Arabidopsis plants.

Sample	-DCMU	+DCMU
(nmoles O_2_^-^ mg Chl^−1^ h^−1^)		
Control	100±10	20±2
ALA-treated	105±11	21±2

Thylakoid membranes were isolated from leaves before the day-break in dark and were suspended at a concentration of 1****mg Chl ml^−1^. The thylakoid membranes were illuminated by incandescent light (500 µmoles photons m^−2^ s^−1^) passed through heat and neutral density filters. O_2_^-^ generation by the thylakoid membranes was monitored as cytochrome C reduction.

### Tolerance of *PORCx* plants to oxidative damage caused by the photodynamic herbicide

**Figure 6:**
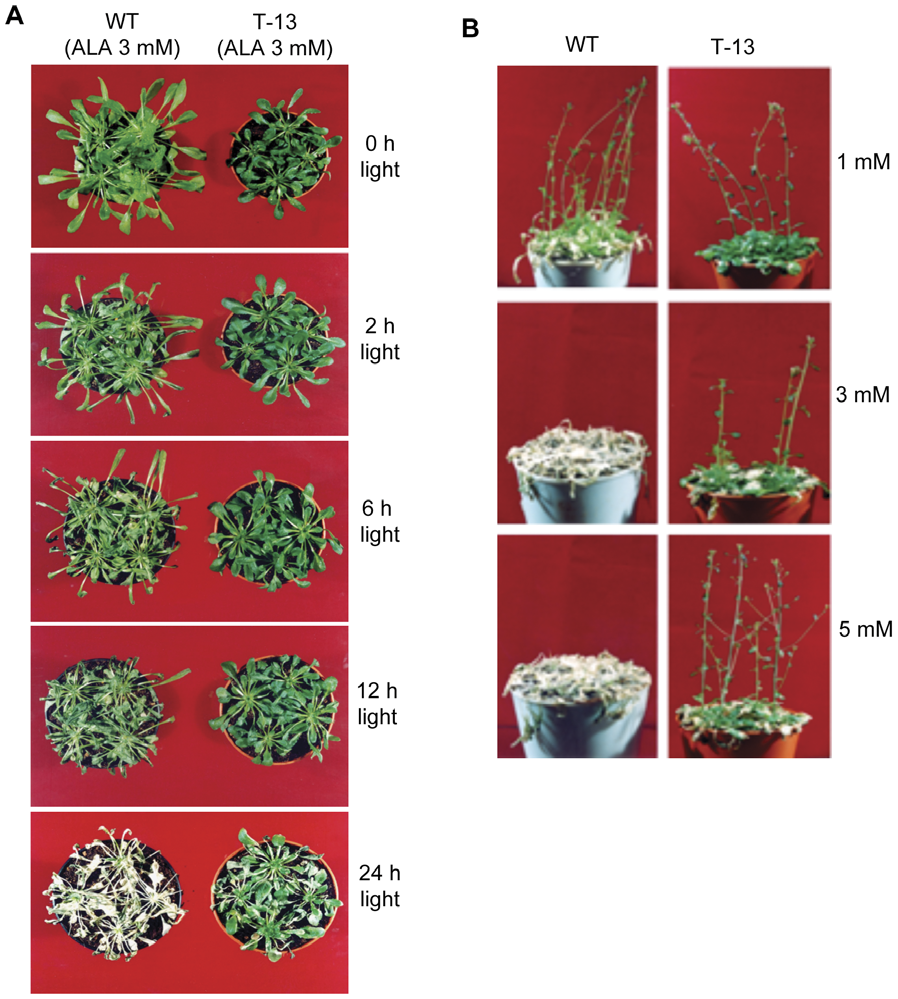
*PORCx* plants are tolerant to ALA-inducd oxidative damage. WT and *PORCx* (T-13) plants were treated with ALA and exposed to light for different time periods. (**A**) Photographs of T-13 plants under ALA-induced oxidative damage. Notice the death of the WT plants after 24 h of light exposure, whereas T-13 plants are slightly damaged. (**B**) Survival of light-exposed T-13 plants treated with different concentration of ALA. Both WT and T-13 plants grown under the same condition as described above were treated with different concentration of ALA (from 1 mM to 5 mM) and their dose dependent tolerance was observed. Notice the WT plants were killed by 3 mM or 5 mM ALA-treatment.

To study the tolerance of *PORCx* plants to ALA-induced oxidative damage, WT and T-13 plants were sprayed with 3 mM ALA, kept in dark for 14 h and then exposed to light (100 µmoles photons m^−2^ s^−1^) for different time periods. The leaves of ALA-treated WT plants started wilting after 2 h of light exposure and they were severely damaged after 6–12 h of illumination (Figure 6A). After 24 h of continuous light exposure WT plants were completely bleached. Under identical conditions *PORCx* plants were tolerant to the ^1^O_2_-induced oxidative stress (Figure 6A).

To understand the dose-dependence of herbicidal action on WT and T-13 plants they were sprayed with 1, 3, 5 or 10 mM ALA and were kept in dark for 14 h to accumulate the photosensitizer Pchlide and were then exposed to light (100 µmoles photons m^−2^ s^−1^) for 10 days (14 h L/10 h D). After 10 days of light exposure, both WT and *PORCx* plants treated with 1 mM of ALA survived. However, the WT plants had more necrotic leaves than that of T-13 plants (Figure 6B). WT plants treated with 3 or 5 mM of ALA perished whereas the T-13 plants survived and produced flowers (Figure 6B). At 10 mM ALA, both WT as well as T-13 plants perished (not shown).

### Fv/Fm, electron transport chain, electrolyte leakage, and MDA content

**Figure 7:**
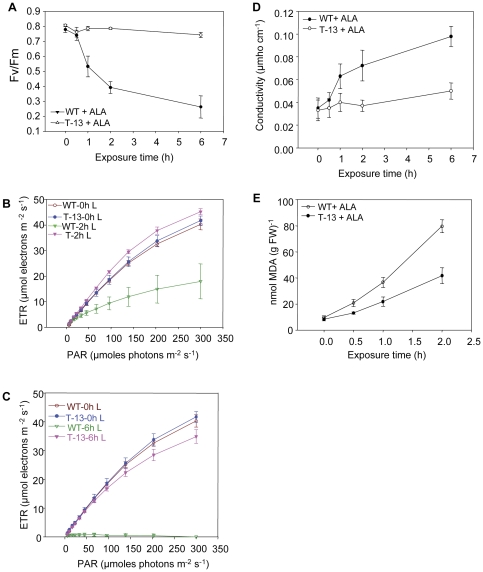
Physiological responses of *PORCx* plants to ALA-mediated oxidative stress. Both WT and T-13 plants were grown and treated with ALA (3 mM) and leaf samples were taken for analysis immediately prior (0 h) or after different duration of light (100 µmoles photons m^−2^ s^−1^) exposure. (**A**) Fv/Fm ratio and electron transport rate (**B, C**) of ALA-treated light exposed WT and T-13 plants as monitored by PAM 2100 fluorometer and values are mean ± SD (n = 20). (**D**) Ion leakage as a measure of damage to plasma membrane in ALA-treated light exposed WT and T-13 plants. (**E**) MDA contents of ALA-treated light exposed WT and T-13 plants. All the above experiments were performed thrice and each data point is the average of 3 replicates. The error bar represents ± SD.

To ascertain if ALA-induced oxidative stress affected photosynthetic quantum yield, Fv/Fm ratio was monitored in ALA-treated WT and *PORCx* plants. The Fv/Fm ratio of dark-incubated ALA-treated WT plants were reduced by 32%, 46%, and 63% respectively after 1 h, 2 h and 6 h of light exposure. Under identical conditions *PORCx* plants had no substantial decrease of Fv/Fm ratio (Figure 7A). There was no change in initial F_0_ fluorescence in WT and *PORCx* plants up to 6 h of light exposure.

The electron transport rate (ETR) of PSII increased in response to photosynthetic active radiation (PAR) (µmoles photons m^−2^ s^−1^) (Figure 7B, C). ALA-sprayed WT plants, exposed to light for 2 h had 55% reduction in ETR and the latter was almost abolished in ALA-sprayed WT plants after 6 h of light exposure (Figure 7B, C). Under identical conditions the ETR of *PORCx* plants was not substantially affected. There was only a partial loss (15%) of ETR of 6 h light exposed *PORCx* plants (Figure 7B, C).

To ascertain if ^1^O_2_ -induced injury to the photosynthetic apparatus resulted in ultimate damage to the plasma membrane and consequent destruction of cellular integrity, changes in the conductivity of the bathing medium of leaf discs excised from ALA-treated and light-exposed WT and T-13 plants were measured. Increase in conductivity of the bathing medium was observed in leaf discs of ALA-treated WT plants within 30 min of light (100 µmoles photons m^−2^ s^−1^) exposure and this increase continued up to 6 h. In comparison to WT, the leaf discs excised from ALA-treated T-13 plants had reduced (50%) ion leakage (Figure 7D).

The loss of plasma membrane integrity was due to increased membrane lipid peroxidation in WT plants. The contents of MDA, the lipid peroxidation product increased in ALA-treated WT plants after 2 h of light treatment. However, in *PORCx* plants MDA production was less i.e., nearly half of that of WT suggesting that membrane lipid peroxidation was substantially reduced in transgenics (Figure 7E). As expected the Fv/Fm ratio, the ETR, electrolyte leakage or MDA production in untreated light-exposed samples or ALA-treated dark incubated samples were not affected.

## Discussion

We show here that overexpression of PORC results in efficient photo-transformation of Pchlide to Chlide and increased synthesis of ALA in *PORCx* plants. The coordinated upregulation of gene/protein expression of several enzymes involved in tetrapyrrole biosynthesis leads to enhanced Chl accumulation that elucidate a regulatory net work of gene expression of tetrapyrrole biosynthetic enzymes. Due to PORC overexpression in Arabidopsis, the light-mediated conversion of Pchlide to Chlide increased in green tissue by 50% over that of WT. In Arabidopsis etiolated tissues, most of the Pchlide was phototransformed by flash illumination because of massive accumulation of PORA protein. However, POR protein abundance becomes substantially smaller in illuminated tissues than the total POR present in etiolated tissues. Therefore, in green tissues complete transformation of Pchlide to Chlide does not occur. Due to PORC overexpression in green tissues phototransformation increased substantially i.e., by 50% (Figure 3A) although there was 1.5-3 fold increase in PORC protein abundance. This could be due to limitation of availability of NADPH and/or functional state of POR enzyme after overexpression. Due to increased photo-transformation of Pchlide and enhanced rate of Chl synthesis, Pchlide and other intermediates of Chl biosynthesis decreased and consequently, generation of ^1^O_2_ was reduced. Therefore, the ^1^O_2_-mediated photo-oxidative damage in high-light-stressed *PORCx* plants was minimal. It was earlier shown that ALA application to plants cause photo-oxidative damage and ultimately kills plants. The present study demonstrates that photo-oxidative damage and ultimate plant death caused by ALA could be substantially minimized by overexpression of PORC that limits the generation of ^1^O_2_. This approach could be biotechnologically exploited further to use ALA as a commercial selective herbicide.

We preferred PORC overexpression over that of its other two isoforms i.e., PORA and PORB [21], [24], [34]–[37], as PORA and PORB are either almost completely or partially down-regulated in light-grown Arabidopsis plants. To the contrary *PORC* expression is induced by light and its transcript and protein abundance increases in response to increase in light-intensity and are predominantly present in matured light-grown green tissues [23], [27]. Lack of PORA either in wild-type seedlings grown under constant far red light (cFR) or in *det340* (de-etiolated) mutant reduced the prolamellar body formation resulting in photooxidative damage to the respective seedlings [34], [38]. Overexpression of either PORA or PORB increases the size of the prolamellar body in cFR-grown wild-type seedlings or in *det340* etiolated seedlings and protects them from oxidative damage [34], [35]. The function of PORB and PORC in greening process of etiolated seedlings has been characterized in knockout mutants (*porb*, *porc*) of *Arabidopsis thaliana* [27], [39]. The *porbporc* double mutant displayed a seedling-lethal *xantha* phenotype [39]. Arabidopsis *porc* T-DNA mutant seedlings had reduced Chl contents as compared to *porb* mutants when exposed to high light [27], [40] that demonstrates the importance of PORC in the regulation of Chl biosynthesis in high light. We have not tested the impact of PORA and PORB overexpression on ALA-induced or high-light-induced oxidative damage. As PORA is severely down regulated in light, its overexpression may not have similar impact as that of PORC in light-grown green plants. However, overexpression of PORB that is only partially down regulated in light, may contribute to tolerance of green plants to oxidative stress similar to that of PORC overexpression albeit to a reduced extent.

ALA is the precursor of Pchlide and Chl [12], [13]. Pchlide regulates its own accumulation via feedback inhibition of ALA synthesis [29], [30]. Therefore, synthesis of ALA, declines within 1 h after transfer of seedlings from light to dark and correlates with an immediate accumulation of Pchlide in darkness [30]. Controlled regulation of ALA synthesis prevents accumulation of tetrpyrrolic metabolic intermediates and avoids photo-oxidative damage. Overnight accumulation of almost similar amounts of Pchlide in the WT and *PORCx* plants, although the latter had enhanced Pchlide biosynthesis potential, demonstrates that regulatory net work is not disturbed due to PORC overexpression. In *PORCx* plants, immediate photo-transformation of Pchlide to Chlide decreased Pchlide contents and consequently released the feed back inhibition that resulted in increased ALA synthesis (Figure 3B) as well as augmented gene/protein expression of the downstream Chl biosynthetic enzymes that elucidates a regulatory net work of gene expression of tetrapyrrole biosynthetic enzymes.

The end product of POR-mediated photo-reaction Chlide synthesized during Chl biosynthetic reaction usually does not generate ^1^O_2_ in plants. This argument stems from the absence of the photo-toxicity of large amounts of Chlide synthesized during greening of plants in the presence of natural sunlight. This is because Chlide synthesized by POR does not accumulate and immediately converted to Chl that binds with Chl-binding protein and transfers its absorbed light energy to the reaction center for its conversion to chemical energy. The synchronized enhanced availability of Chl-binding protein, as evident from increased LHCII proteins in *PORCx* plants (Figure 1E) prevents the accumulation of PORC photo-reduction product Chlide or free Chls. This reduces the generation of ^1^O_2_ and photodynamic damage by Chlide. In the same vein it is previously shown that PORA and/or PORB overexpression although results in higher Chlide formation does not lead to photo-oxidative stress caused by far-red light [36]. However, Chlide formed during Chl degradation process because of senescence or stress accumulates in the cell for a longer time and has the potential to cause photo-toxicity [41]. Exogenous application of ALA increased the accumulation of Chl that led to augmented synthesis of LHC II apoproteins [42]. Binding of Chls to the LHC II apoproteins is necessary to stabilize the LHC II apoproteins; when there is no Chls i.e., in etioplasts LHC II apoproteins gets degraded by the proteases [43]. Similarly, levulinic acid that inhibits Chl synthesis, also restrains the LHC II apoprotein accumulation [44]. Therefore, transcriptional and translational control of LHC II apoproteins amount may not be sufficient to maintain the stoichiometry of apoproteins to Chl. Binding of Chl by apoproteins and breakdown of excess apoproteins could be an important mechanism for the turnover of LHC II [43].

The ^1^O_2_ produced in high light is often dissipated via specific carotenoids that are in close proximity to Chl in light-harvesting chlorophyll-complexes [16]. However, increase in the carotenoid pool size may not be always enough to reduce ^1^O_2_ produced from Pchlide or other non-esterified Chl intermediates i.e., PPIX or MP(E), because these intermediates are not associated with light-harvesting chlorophyll-protein complexes and hence are not connected to the reaction centre. For quenching of Chl precursors triplet states to occur, the Chl precursors and carotenoids need to be in very close proximity for fast (excitation) energy transfer and efficient photoprotection. Although, some of the carotenoids are partially present in the lipid bilayer, a lot more are located in the pigment-protein complexes [17], [45] and they are spatially too far from Chl biosynthesis precursors to quench their triplet states. Water soluble chlorophyll protein (WSCP) also reduces ^1^O_2_ production from free photosensitized Chl molecules [46]. The tetrameric WSCP-Chl complex encloses Chl molecules in a water-tight cavity and that enclosure reduces the chance of direct contact between the Chl molecules and O_2_. Although, WSCP binds to Chl biosynthetic intermediate Mg-protoporphyrin IX, the binding does not induce the tetramerization of WSCP [47] and consequently a water-tight cavity is not produced. Therefore, WSCP is likely to fail to prevent the production of ^1^O_2_ from Chl biosynthetic intermediates.

Antisense expression of some of the genes involved in Chl biosynthesis pathway [6], [48], [49] leads to the accumulation of Chl biosynthesis intermediates leading to light-induced oxidative damage. Plants treated with ALA also get damaged by sun light because of overaccumulated Pchlide [2], [4], [5], [8]. To study the real effectiveness of PORC overexpression to minimize the ^1^O_2_ generation in light, our previous approach to over-produce Pchlide by spraying the plants with ALA [4] was probed further. We demonstrated that PORC overexpression could protect plants from ALA-induced damage and death. For phototransformation of Pchlide to Chlide, it is prerequisite for Pchlide to bind to POR and NADPH to form a multimeric ternary complex. Without binding of Pchlide with POR and NADPH, phototransformation of Pchlide to Chlide can not occur. ALA-treated *PORCx* plants had reduced accumulation of the photosensitizer Pchlide in light than that of WT plants due to increased photo-transformation of Pchlide bound to overexpressed PORC after 10 min of light exposure. The minimal accumulation of Pchlide resulted in reduced light-activated generation of ^1^O_2_ than that of WT. Consequently, when exposed to light *PORCx* plants had minimal damage to their leaves, their various physiological functions were not much affected and the plants survived and bolted (Figures 6 and 7), whereas ALA-treated WT plants were badly affected and completely bleached by oxidative damage. A particular potential hazard of the formation of ^1^O_2_ arises from its role in initiating lipid peroxidation reaction that consumes chloroplast membrane lipids [4], [5], [50]. In ALA-treated WT plants, the kinetics of loss of quantum efficiency of PS II of the thylakoid membranes matched well with that of electrolyte leakage due to damage to the plasma membrane (compare Figure 7A and 7D, F). We also observed similar kind of results in high-light treated WT plants where the extent of generation of MDA (Figure 4G), a product of lipid peroxidation, correlates with relative amounts of ^1^O_2_ production demonstrating the pivotal role of ^1^O_2_ in membrane lipid peroxidation and oxidative stress. This demonstrates that although ^1^O_2_ is a short-lived species generated inside the plastid, it could potentially migrate to plasma membrane causing lipid peroxidation and injury. This conclusion is consistent with microscopically visualized movement of ^1^O_2_ across the cell [51]. The tetrapyrroles are localized in the stroma, envelope and thylakoid membranes of chloroplasts [52]. The ^1^O_2_ generated in the envelope membranes could also potentially diffuse to plasma membrane to damage the cell membrane or to the nucleus to act as a signalling molecule to induce programmed cell death.

Our results demonstrate that ALA could be used as selective commercial herbicide. ALA could be produced from levulinic acid by addition of amino group at C5 position [53]. The precursor of ALA, i.e., levulinic acid could be produced cheaply from cellulosic material of organic waste in a chemical reactor [54]. Biotechnologically, ALA (20 mM) could also be produced cheaply by extra-cellular secretion of *E*.*coli* overexpressing ALA synthase i.e., *hemA* from *Bradyrhizobium japonicum* expressed under the control of T7 promoter [55].

## Materials and Methods

### Plant materials and growth conditions

The *Arabidopsis thaliana* ecotype Columbia (Wild-type: WT) and PORC-overexpressing lines (*PORCx*: T-9, T-12, T-13) were grown in a growth chamber (Conviron, Canada) under cool-white-fluorescent + incandescent light (100 μmoles photons m^−2^ s^−1^), and a 14 h light/10 h dark photoperiod at 22 ^0^C ± 2 ^0^C. For experiments related to ALA treatment WT and *PORCx* plants were grown for 28–32 days in the above -mentioned condition. For different light treatment, plants were grown for 22–24 days in the growth chamber and subsequently exposed to high-light (HL) (350 μmoles photons m^−2^ s^−1^, 16 h light/8 h dark) and low-light (LL) (50 μmoles photons m^−2^ s^−1^, 16 h light/8 h dark) for additional 6–7 days at 22°C±2°C.

### ALA treatment

Each plastic pot, having 4 plants, was sprayed with 15 ml of aqueous ALA solution (pH 4.9) under safe light as described previously [4]. Control plants were sprayed with distilled water (pH 4.9). After ALA/distilled water treatment, the plants were kept in the dark for 14 h and subsequently exposed to light (100 μmoles photons m^−2^ s^−1^) for different time period.

### Transformation of *AtPORC* in Arabidospsis

The recombinant pGEMT-Easy plasmid containing the full length *PORC* cDNA [25] was EcoRI digested and the digested product was cloned into the modified pCAMBIA 1304 binary vector at the EcoRI site in the sense orientation under the control of CaMV 35S promoter fused to the omega translational enhancer (CaMV35S-Ω-*AtPORC*-polyA). The latter cassette was taken from the pSH9 [56]. pCAMBIA 1304 binary vector was modified as described before [57]. The hygromycin (*hpt*) marker gene was replaced with kanamycin (*nptII*) gene. The pCAMBIA1304::*AtPORC* construct was introduced into *Agrobacterium tumefaciens* strain GV3101 and used to transform *Arabidopsis thaliana* (Col) by vacuum infiltration method [58]. Primary transformants were selected on half-MS agar medium containing 50 mg/L kanamycin and were grown to T3 generation. T3 homozygous lines were used for further analysis.

### Semi-quantitative RT-PCR analysis

Total RNA from Arabidopsis plant was isolated using TRIzol reagent (Invitrogen, USA) and treated with 5 U of RNase free DNase I (Promega, USA) to make sure that there is no contamination of DNA. For reverse transcription (RT)–PCR, first-strand cDNA was synthesized using 2 µg of total RNA, oligo (dT) primer, and AMV reverse transcriptase (Promega, USA) in a 50 µL reaction. After synthesis, the cDNA was diluted 1∶10, and 2 µL of cDNA was used as a template for PCR amplification in a 25 µL reaction mixture using gene specific primers. Reaction contained selected couples of following gene specific primers: *GluTR* F, 5’-atg aac aag aag tga gga aaa c-3’, *GluTR* R, 5’-acc ttt gct cta atc ttc tcc t-3’; *GSAT* F, 5’-gag cga cac aga gaa gtt tgg-3’, *GSAT* R, 5’-cct act cag tac cct ctc agc-3’; *UROD* F, 5’-ccg gtg tgg atg ttg tga gc-3’, *UROD* R, 5’-agt atc atg aat ccg gct tgt g-3’; *PPOX* F, 5’-gac acg gct aaa tca tct cta ac-3’, *PPOX* R, 5’-cgg gat cct gtt cag tgg ccg gtg gac ca-3’; *CHLD* F, 5-gat acc gag aac aag ttt gtt tc-3’, *CHLD* R, 5’-cgg gat cca agc ttt caa gaa ttc ttc aga tca g-3’; *CHLP* F, 5’-gca tgg cga cga cgg tta ca-3’, *CHLP* R, 5’-gct taa aca cta agc ttc tca atc tc-3’; *CHS* F, 5’-atg gtg atg gct ggt gct tc-3’, *CHS* R, 5’-tta gag agg aac gct gtg caa g-3’; *ACT1* F, 5’-atg gct gat ggt gaa gac att-3’, *ACT1* R, 5’-tca gaa gca ctt cct gtg aac a-3’. To ensure linearity of the reaction, the minimum number of cycles needed to visualize the transcripts was first determined (26 cycles for *GLUTR*, *GSAT*, *UROD* and *ACT1* and 29 cycles for *PPOX*, *CHLD*, *CHS* and *CHLP*) and runs were repeated for three times using independently treated samples. *AtACT1* was used as an internal control. Fifteen µl of PCR products were separated on 1% Agarose Tris-acetate EDTA gel containing ethidium bromide, photographed and their signal intensities were quantified using the Alpha Imager 3400.

### Estimation of 5-aminolevulinic acid (ALA)

Leaves (200 mg), harvested from WT and *PORCx* (T-12 and T-13) plants were incubated in the presence of 50 mM levulinic acid dissolved in 50 mM MES, pH 7.0. Batches of 200 mg leaves were kept in levulinic acid under cool white fluorescence light (30 µmoles photons m^−2^ s^−1^) for 6 h in both light and dark. Leaves were hand homogenized under green safelight in 5 ml of ice cold 4% trichloroacetic acid and centrifuged at 10,000 rpm for 10 min at 4 ^0^C. Supernatant was taken for ALA estimation [59]. Results were expressed as net ALA synthesis (ALA synthesis in dark subtracted from ALA synthesis in light).

### Isolation of plastid proteins and Western blot analysis

Plastids were isolated from Arabidopsis leaves by homogenizing about 4 g of tissues in 40 ml of isolation buffer containing 50 mM Hepes, pH 7.5, 400 mM sucrose, 1 mM EDTA, 1 mM MgCl_2_, and 2 mM isoascorbate at 4 ^0^C under safe green light [59]. Thirty micrograms of plastid proteins were electrophoretically transferred to nitrocellulose membrane [57]. The PORC specific monoclonal antibody (kindly provided by T. Masuda, University of Tokyo, Japan), the LHC II polyclonal antibody (kindly provided by R Bassi, University of Verona, Italy) and the RbcL polyclonal antibody (kindly provided by Ayumi Tanaka, Hokkaido University, Japan) were used at a dilution 1/1000, 1/5000 and 1∶20000 respectively and blots were immuno-detected using the ECL system (Amersham–Pharmacia, USA). The other polyclonal antisera used in this study were described elsewhere and the blots were immuno-detected using 5-bromo-4-chloro-3-indolyl phosphate and nitro blue tetrazolium [60] and quantified using an Alpha Imager 3400. All the western blots were repeated thrice.

### Pigment estimation

Chl and carotenoid contents were estimated in 80% acetone as described elsewhere [61], [62]. The estimation of the Chl biosynthesis intermediates was done spectrofluorometrically using a photon-counting SLM 8000 spectrofluorometer in the ratio mode as described elsewhere [59], [63].

### Photo-transformation of protochlorophyllide to chlorophyllide

Four-week-old light-grown (100 µmoles photons m^−2^ s^−1^, 14 h light/10 h dark photoperiod) WT and *PORCx* plants were kept in dark for 14 h followed by 10 min light exposure (100 µmoles photons m^−2^ s^−1^) and then the photo-transformation of Pchlide was measured. Fifty mg leaf tissues were taken from 14 h dark incubated and 10 min light-illuminated plants and processed for Pchlide measurement under safe green light as described before [58], [62]. When the plants (28–32 days old plants grown under 14 h light/10 h dark photoperiod) were treated with ALA, 15 ml of 3 mM of ALA was sprayed to both WT and *PORCx* plants under safe green light and ALA sprayed plants were kept in dark for 14 h. After 14 h of dark incubation plants were exposed to light (100 μmoles photons m^−2^ s^−1^) for 10 min and leaves (50 mg) were harvested and their Pchlide contents were monitored as described before [59], [63]. The percent phototransformation of Pchlide to Chlide in different samples was calculated as [(Pchlide content before phototransformation) - (Pchlide content after phototransformation)]/Pchlide content before phototransformation) x 100.

### ^1^O_2_ measurement

The production of ^1^O_2_ from thylakoid membranes was determined in terms N, N-dimethyl-p-nitrosoaniline (RNO) bleaching using histidine as a trap of ^1^O_2_ [5]. Thylakoids (100 μg Chl ml^−1^) were incubated with RNO solution (300 μM RNO in 10 mM histidine) in one tube in the dark, whereas another tube was illuminated with light (500 μmol photons m^−2^ s^−1^) for different time periods during the assay. Samples were centrifuged (12,000 × g) after dark or light treatment and their absorbance was read at 440 nm in a spectrophotometer (Shimadzu, UV160). The dark samples were used as reference.

### O_2_^-^ measurement

Intact chloroplasts were isolated in diffuse green safe light from leaves (5 g) of WT control and ALA-treated plants incubated in dark by centrifugation through 40% percoll gradient and thylakoid membranes were prepared by osmotic lysis in TE buffer in dark [64]. This procedure of thylakoid membrane isolation from intact chloroplasts eliminated mitochondrial contamination and consequent presence of cytochrome C oxidase. To remove stromal superoxide dismutase the thylakoid membranes were suspended in 50 mM phosphate buffer (pH 7.8) containing 1 mM EDTA kept for 1 h at 4 ^0^C, and then centrifuged at 3000 g for 10 min. This washing procedure was repeated twice. The production of O_2_^-^ was determined spectrophotometrically by monitoring cytochrome c reduction at 550 nm using an extinction coefficient of 19 mM cm^−1^ [65]. The 3 ml reaction mixture consisted of 50 mM phosphate buffer (pH 7.8), 10 mM NaCl and 20 mM ferricytochrome C. Thylakoids containing 50 mg Chl were added to the reaction mixture. The rate of reaction was determined from the initial absorbance increase 1 minute after illumination. Under the above experimental conditions an increase in 0.01 A at 550 nm equals the production of 1.05 nmoles of O_2_^-^.

### Anthocyanin measurement, estimation of lipid peroxide and electrolyte leakage

The anthocyanin estimation was carried out as done before [66]. The amount of lipid peroxidation products was estimated as described [67]. For determination of ion leakage, leaf discs (12 in number, 8 mm diameter) from both control and treated plants were kept in petriplates containing 20 ml deionised water under constant shaking (60 rpm) for 6 h. After specific interval of time the extent of solute leakage (conductivity) in the bathing medium was monitored using a conductivity meter.

### Fv/Fm and electron transport rate (ETR) measurement

Fv/Fm and ETR of attached leaves that had been dark adapted for 20 min was measured at room temperature by Walz PAM-2100 Chl fluorometer [68].
